# Associations of Body Dissatisfaction With Lifestyle Behaviors and Socio-Demographic Factors Among Saudi Females Attending Fitness Centers

**DOI:** 10.3389/fpsyg.2021.611472

**Published:** 2021-02-11

**Authors:** Nada M. Albawardi, Abeer A. AlTamimi, Mezna A. AlMarzooqi, Lama Alrasheed, Hazzaa M. Al-Hazzaa

**Affiliations:** ^1^Lifestyle and Health Research Center, Health Sciences Research Center, Princess Nourah Bint Abdulrahman University, Riyadh, Saudi Arabia; ^2^Health Sciences Research Center, Princess Nourah Bint Abdulrahman University, Riyadh, Saudi Arabia; ^3^Department of Community Health Sciences, College of Applied Medical Sciences, King Saud University, Riyadh, Saudi Arabia

**Keywords:** body image, perception, body mass index, females, obesity, lifestyle behaviors, Saudi Arabia, body image dissatisfaction

## Abstract

**Objective:**

To examine body image perception and the associations of body dissatisfaction (BD) with socio-demographic and lifestyle factors among Saudi women attending fitness centers in Riyadh.

**Methods:**

Saudi females aged 16 years and older were recruited from 12 randomly selected fitness centers in Riyadh, using stratified clustered sampling technique (*n* = 460). Height and weight were measured to calculate actual body mass index (BMI). A previously validated instrument was used to collect socio-demographic and lifestyle variables including physical activity (PA), sedentary behaviors, sleep and dietary habits. Stunkard Figure Rating Scale silhouettes were used to assess perceived and desired body shape.

**Results:**

The participants had a mean (SD) age of 29.2 (8.2). The majority were not married (57%), with no children (66%) and had college degrees (78%). While 63% were overweight or obese, nearly 40% of women underestimated their perceived body shape. The majority of respondents (87%) were dissatisfied with their body shape including 68% of normal weight women. Females who had BD were significantly older, had higher BMI, reported more weight loss attempt and had expended less time in vigorous (*p* = 0.033) and total (*p* = 0.042) PA than those who were satisfied with their body shape. However, when adjusting for socio-demographic variables, logistic regression analysis revealed significant associations of BD with higher BMI, shorter membership duration of fitness club, and reduced dairy products and energy drinks consumption.

**Conclusion:**

Except for BMI and decreased dairy products and energy drinks consumption, many lifestyle behaviors did not associate with BD among Saudi females attending fitness centers. The findings can inform healthcare providers when intervention strategy is implicated for females with BD. Future studies should compare the associations of BD with lifestyle behaviors between males and females attending fitness centers and seeking weight loss.

## Introduction

The escalating public health concerns relating to obesity and its associated health consequences have led to a plethora of interventions designed to address the growing risk factors of physical inactivity, malnutrition, and eating disorders. These interventions include strategies to affect positive behavioral changes using both conscious, decision-based models as well as models that address non-conscious processes (Rejeski and Fanning, 2019). The effectiveness of such programs is often moderated by a number of variables including psychosocial, biological and environmental factors (Rejeski and Fanning, 2019). Among the psychosocial factors; body image (BI) perception and body dissatisfaction (BD) have been shown to be associated with obesity (Anderson et al., 2002; Duong and Roberts, 2014; Jun and Choi, 2014), physical activity (PA) (Hausenblas and Fallon, 2006), dietary habits (Fan and Jin, 2015), eating disorders (Lawler and Nixon, 2011), and depression (Paans et al., 2018). BI is a multidimensional construct encompassing perceptual, attitudinal, and behavioral dimensions. Body image perception (BIP) is a component of the perceptual and behavioral dimension and relates to how one sees and describes their body in relation to body size, shape, and weight (Sabiston et al., 2019). BD is a component of the attitudinal dimension and refers to an undesirable subjective assessment of one’s body often described as when desired BI does not match with the perceived BI.

Inaccurate perceptions of body size (BIP) are common particularly among children and adolescents (Neves et al., 2017). In a study of nearly 30,000 children and adolescents in Taiwan (Hsu et al., 2016), the prevalence of body weight misperception was 43.2% (26.4% overestimation and 16.8% underestimation). Similarly, in a sample of 4979 adolescents in the in the United Kingdom, 39% of those overweight/obese (47% of boys, 32% of girls) underestimated themselves as “about the right weight” or even “too light” (Jackson et al., 2015). Likewise, over 45% of overweight/obese subjects from a sample of 660 adolescents from the United Arab Emirates believed they were normal weight (Musaiger et al., 2012). While fewer studies report on the accuracy of adults body weight estimations, misperceptions clearly exist. Among 1,295 Dutch older adults, only 4.4% of obese men and 12.3% of obese women accurately perceived their body weight status (Monteagudo et al., 2015). Similarly, in a large sample of overweight/obese young adults in the United States, 20% of females and 48% of males under-estimated their weight (Sonneville et al., 2016). Saudi college aged women with overweight/obesity also underestimated their actual body mass index (BMI) by 8.5% (–2.5 ± 2.5 kg/m2), while those without overweight/obesity slightly overestimated their BMI by 1.9% (Albeeybe et al., 2018).

Irrespective of actual weight status, both over and underestimation have often been found to negatively affect weight management and PA in adolescents (Jankauskiene and Baceviciene, 2019) and adults (Voelker et al., 2015). In a review of 74 studies on weight perception and associated weight management, Haynes et al. (2018) concluded that perceived overweight was not reliably associated with increased PA or healthy eating and was associated with greater disordered eating in some groups. Overestimation, while increasing the number of attempts at weight loss, has consistently been associated with increased likelihood of engaging in health compromising behaviors (Fan and Jin, 2015; Robinson et al., 2015). In addition, both over- and under-estimation of body weight have been associated with unhealthy weight control behaviors and depressive mood (Jankauskiene and Baceviciene, 2019). In a study of over 4000 Canadian adolescents, those perceiving themselves as overweight or obese had lower odds (OR: 0.59; 95% CI: 0.42–0.81) of adherence to PA recommendations compared with those who felt they were about the right weight (Sampasa-Kanyinga et al., 2017). Several longitudinal studies have also shown that perceiving oneself as overweight, regardless of actual size, increased the risk of future weight gain (Robinson et al., 2015; Haynes et al., 2018; Feng and Wilson, 2019). On the other hand, accurate estimation of excess weight was observed to have a positive effect on weight management in a 13-year follow-up of obese women who perceived themselves as too large and showed less weight gain over time than those who underestimated their weight (Lynch et al., 2009). In addition decreased awareness of actual weight status may lead some to be less aware of their risk for negative health consequences (Miller et al., 2008). Thus it appears that body weight estimation may play both a negative and positive role in adopting healthy lifestyle behaviors (Jankauskiene and Baceviciene, 2019).

The health consequences that may result from the unique interaction of age, body mass, and BD are particularly relevant as correlates, precursors, and consequences of PA (Sabiston et al., 2019). A positive BI, rather than BMI, is often a stronger predictor of PA engagement (Kantanista et al., 2015; Sampasa-Kanyinga et al., 2017). While BD is associated with lower PA, and dysfunctional exercise (Sampasa-Kanyinga et al., 2017; Jankauskiene and Baceviciene, 2019), participating in exercise has been shown to have a positive impact on BI (Fernández-Bustos et al., 2019). A meta-analysis on the effect of exercise on BI found exercisers had a more positive BI than non-exercisers (Hausenblas and Fallon, 2006). Exercise interventions also resulted in improved BI scores post-intervention compared to controls (Hausenblas and Fallon, 2006). Positive effects of engaging in PA on BI, with and without changes in body composition, are consistently demonstrated in the literature (Voelker et al., 2015).

Over the past decade, there has been accumulating evidence showing that body weight concerns and dissatisfaction are fairly prevalent among young Arab women. A recently published research involving young Arab females from five Arab countries including Bahrain, Egypt, Jordan, Oman, and Syria revealed that one third of the females were dissatisfied with their body weight status (Musaiger, 2014). Similarly Saudi females reported a notably higher proportion of BID (33.5%) than males (21.4%) (Al-Otaibi et al., 2013). Such findings may call attention to the sociocultural influences and media pressure that emphasized body thinness among Saudi young females (Albeeybe et al., 2018).

Few studies have investigated the associations between BI perception, BD and lifestyle variables. In particular, none of the previous studies had examined the associations of BD and lifestyle behaviors among females engaging in physical activities while mostly seeking body weight loss. Local studies are scarce and either did not focus primarily on the association of BI and lifestyle, used small sample sizes or did not use validated PA or BI measures (Ben-Ammar and Al-Holy, 2013; Alhussaini et al., 2018). In addition, due to the recent social and political changes in Saudi Arabia, there has been a surge in female fitness centers and increased engagement of females in PA, especially those with excess body weight (AlTamimi et al., 2020). Consequently, studying females participating in fitness centers may help in understanding the associations between body satisfaction or dissatisfaction and lifestyle behaviors and socio-demographic factors. Such approach may enhance our understanding of the different factors that promote body satisfaction in females and is an important tool to facilitating the adoption of healthy lifestyle behaviors. Therefore, the aim of the present study is to investigate the status of BI perception and to explore the associations of BD with demographics, PA, screen time, dietary habits and sleep among adult females attending fitness centers in Riyadh, the capital of Saudi Arabia.

## Materials and Methods

### Participants

Twelve fitness centers in Riyadh, Saudi Arabia were randomly selected, using stratified (geographical locations) clustered sampling technique. Saudi females aged 16 years and older without any physical impairment were recruited from these centers using a predetermined sample size of 384 participants. The sample proportion was within 0.05 of the population proportion with a 95% confidence level. The population proportion was assumed to be at 0.50, as this proportion yields the maximal possible sample size required. An additional 20% for clustered design effect and missing data was added resulting in a target sample size of 460 females. All female participants in the sampled fitness center were approached during the data collection period and asked to take part in the study. Visits to the fitness centers included random days during the week and on weekends. Ethical approval was attained from the ethical committee at the Health Sciences Research Center, Princess Nourah University (IRB Log Number: 18-0222). Written consent form was obtained from each of the participating females.

### Anthropometric Measurements

Body weight was measured to the nearest 100 g, with minimal clothing and without shoes, using a calibrated portable medical scale (Seca scale model 770, Seca, Hamburg, Germany). Height was measured to the nearest cm with the subject in the full standing position without shoes using calibrated portable measuring rod. BMI was calculated as body weight in kg divided by squared height in meters and then classified into “underweight,” “normal,” overweight,” and “obese” according to World Health Organization (WHO) criteria (World Health Organization, 2000).

### Perceived and Desired Body Image Assessment

Stunkard Figure Rating Scale (FRS) nine silhouette figures were used to assess perceived and desired BI (Stunkard et al., 1983). The perceived BI scores were classified into BMI categories according to Bulik et al. (2001), who established the equivalence between each silhouette and BMI in a study of 16,728 American women and 11, 366 men. Silhouettes were categorized as underweight (silhouette 1), normal weight (silhouette 2–4), overweight (silhouette 5 and 6), and obese (silhouette 7–9). The discrepancy between the actual BMI category and the BMI category corresponding to the perceived BI score represents the accuracy of the respondents BI perception. Accuracy of BIP was stratified into “overestimation” (perceived > actual BMI category), “accurate” (perceived = actual BMI category), and “underestimation” (perceived < actual BMI category).

Second, the discrepancy between perceived and desired BI scores was calculated to represent the individual’s satisfaction with their BI. Respondents were categorized as “dissatisfied” if they desired a larger or smaller figure than their perceived image and “satisfied” if the perceived score was the same as the desired score.

### Physical Activity, Screen Time, and Sleep Duration Assessment

The Arab Teen Lifestyle Study (ATLS) questionnaire was used to collect data related to PA, screen time, dietary habits, and sleep duration (Al-Hazzaa and Musaiger, 2011; Al-Hazzaa et al., 2011a). The questionnaire collects information on the frequency, duration, and intensity of several domains of activity including transport, household, fitness and sporting and leisure-time activities of light-, moderate-, and vigorous-intensity during a typical week. PA time per week was converted to activity energy expenditure values in METs-min/week, based on compendium of PA for adults (Ainsworth et al., 2011). The minimum activity energy expenditure was computed as 600 METs-min/week [equivalent to 150 min of moderate (four METs) per week]. In addition, participants reported on the typical time in hours spent per day on screen activities, including television (TV) viewing, video games, and computer and internet use during both weekdays and weekends. Low and high screen time cut-off values were based on below or above 2 h of screen viewing per day. The typical sleep durations at night in hours spent on weekdays and weekends were also reported by the participants. Insufficient sleep was defined as sleeping less than 7 h per night, according to the definition of the National Sleep Foundation for adults (Hirshkowitz et al., 2015). Previous studies have shown this questionnaire to be valid and reliable for assessing PA and other lifestyle habits in adolescents and young adults (Al-Hazzaa and Al-Ahmidi, 2003; Al-Hazzaa et al., 2011b).

### Data and Statistical Analysis

Data was entered into an SPSS data file, checked, cleaned, and analyzed using SPSS program, version 22 (IBM, Chicago, IL, United States). Descriptive statistics were calculated and presented as means and standard deviations (SD) or frequencies and proportions. Bivariate analysis comparing the participants who were “satisfied” with their shape with those who were “dissatisfied and desired a smaller shape” was conducted using Chi-Square or *T*-tests as appropriate. Partial correlation coefficients (Pearson), while controlling for age, was used to test the relationships between perceived BI, desired BI and BD with selected lifestyle behaviors. Multivariable logistic regression were used to examine the associations between BI satisfaction and dissatisfaction with levels of accuracy of BI perception (underestimated, accurately estimated, and overestimated BI), socio-demographic characteristics and engagement in lifestyle behaviors, specifically related to dietary intake, PA, and sleep. In all of the tests performed, we used alpha level of <0.05 as the level of significance.

## Results

After approaching 517 female participants during both weekdays and weekends, 460 agreed to take part in the study and this number constituted the final sample size, constituting a response rate of 89%. Mean age (SD) of the sampled females was 29.2 (8.2) years ranging from 16 to 63 years. Body weight and height averaged 69.7 (14.7) kg and 158.7 (5.5) cm, respectively, while the mean (SD) of BMI was 27.6 (5.4) kg/m^2^ with 63% overweight or obesity prevalence. The majority of respondents were not married (57%), did not have children (66%) and had college degrees (78%). Only 11% of women reported being “satisfied” with their body shape. The majority of women, 79%, was “dissatisfied” and desired a smaller shape, while only 5% desired a larger shape. The mean perceived and desired BI scores, based on FRS were 4.5 (1.6) and 2.8 (0.91), respectively. This means that the differences between perceived and desired BI scores was 1.7 (1.4) or 37.8%. BMI corresponding to perceived and desired BI scores, based on BMI values corresponding to desired BI scores reported by Bulik et al. (2001) were 25.5 (4.9) and 20.8 (1.8), respectively. This is a difference of 18.4%, while the difference between the actual BMI and BMI corresponding to perceived BI score was 7.9%.

The socio-demographic characteristics of women who were “satisfied” and those “dissatisfied and desiring a smaller shape” are reported in Table 1. The mean BMI of those who were dissatisfied (28.7 ± 5.1) was significantly higher than those who were satisfied with their body shape (23.3 ± 3.2, *p* < 0.001). The proportion of women with BD increased significantly with BMI categories (normal-61%, overweight-95%, obese- 98%, *p* < 0.001). The participants’ perception of their body shape was accurate for 52% of the respondents, while 40% underestimated their body shape, choosing a perceived body shape lower than that corresponding to their actual BMI. Dissatisfaction was significantly associated with weight loss attempts, with the proportion of women reporting BD increasing with increased weight loss attempts (*p* < 0.001). Duration of club membership was also significantly associated with BD (*p* = 0.028).

**TABLE 1:** Association of body image dissatisfaction with socio-demographic variables and accuracy of perception of body shape among Saudi women attending fitness clubs in Riyadh, Saudi Arabia.

Variable	All	Body Image		*p*-Value^2^
		Satisfied	Dissatisfied^1^	
All	414 (100%)	52 (12.6%)	362 (87.4%)	
Age (Mean ± SD)	29.5 ± 8.05	28.3 ± 7.01	29.6 ± 8.19	0.304
**Age category (%)**				0.028
<30 years	214 (57.5%)	34 (15.9%)	180 (84.1%)	
≥30 years	158 (42.5%)	13 (8.2%)	145 (91.8%)	
BMI (Mean ± SD)	28.0 ± 5.3	23.3 ± 3.2	28.7 ± 5.1	<0.001
**BMI category (%)**				<0.001
Underweight	1 (0.2%)	0 (0%)	1 (100%)	
Normal	136 (33.1%)	43 (31.6%)	93 (68.4%)	
Overweight	153 (37.2%)	6 (3.9%)	147 (96.1%)	
Obese	121 (29.4%)	2 (1.7%)	119 (98.3%)	
**Marital status (%)**				0.258
Not Married	224 (54.2%)	32 (14.3%)	192 (85.7%)	
Married	189 (45.8%)	20 (10.6%)	169 (89.4%)	
**Number of children (%)**				0.135
No children	262 (63.3%)	39 (14.9%)	223 (85.1%)	
1–2	54 (13%)	6 (11.1%)	48 (88.9%)	
3+	98 (23.7%)	7 (7.1%)	91 (92.9%)	
**Educational level (%)**				0.232
High school or less	91 (22.2%)	8 (8.8%)	83 (91.2%)	
College and more	319 (77.8%)	43 (13.5%)	276 (86.5%)	
Father education (%)	0.156			
High school or less	163 (39.8%)	16 (9.8%)	147 (90.2%)	
College and more	247 (60.2%)	36 (14.6%)	211 (85.4%)	
**Mother education (%)**				0.144
High school or less	252 (62.2%)	27 (10.7%)	225 (89.3%)	
College and more	153 (37.8%)	24 (15.7%)	129 (84.3%)	
**Family income (SR)^3^ (%)**				0.118
15,000 or less	159 (40.7%)	14 (8.8%)	145 (91.2%)	
15,001–25,000	123 (31.5%)	20 (16.3%)	103 (83.7%)	
>25,000	109 (27.9%)	17 (15.6%)	92 (84.4%)	
**Club membership (%)**				0.028
Less than a year	284 (77.4%)	27 (9.5%)	257 (90.5%)	
1–2 years	58 (15.8%)	12 (20.7%)	46 (79.3%)	
3–4 years	16 (4.4%)	4 (25%)	12 (75%)	
5 years or more	9 (2.5%)	2 (22.2%)	7 (77.8%)	
**Weight loss attempts (%)**				<0.001
No attempt	83 (20%)	24 (28.9%)	59 (71.1%)	
1–2 attempts	127 (30.7%)	16 (12.6%)	111 (87.4%)	
3 attempts or more	204 (49.3%)	12 (5.9%)	192 (94.1%)	
**Body image perception (%)^4^**				0.750
Overestimate	28 (6.8%)	1 (3.6%)	27 (96.4%)	
Accurate	216 (52.2%)	42 (19.4%)	174 (80.6%)	
Underestimate	167 (40.3%)	8 (4.8%)	159 (95.2%)	

*^1^Desired shape < perceived shape. ^2^Chi Squares tests for the differences in proportions, *T*-test for comparison of means (SD). ^3^US $ = 3.75 SR. ^4^Body image perception: Overestimated = actual BMI category < BMI category of perceived body shape. Accurate = actual BMI category < BMI category of perceived body shape. Underestimated = actual BMI category > BMI category of perceived body shape.*

Table 2 displays the association of lifestyle variables with subjects who were “satisfied” and those who were “dissatisfied and desired a smaller shape.” The majority of the respondents screen time was over 2 h per day (89%), however over 92% participated in PA for 150 min per week or more. The “satisfied” group had significantly higher energy expenditure in METs-min/week of vigorous and total activity (*p* = 0.033, 0.042, respectively). The average sleep time reported was 6.71 ± 1.57 h per day with 46 % of women sleeping 7 h or more per night. The mean frequency of eating breakfast, fruits, vegetables and dairy products was less than five times per week. Unhealthy foods were consumed an average of less than two times per week except for chocolates/candy (mean 2.7 ± 2.3).

**TABLE 2:** Association of body image dissatisfaction with lifestyle variables among Saudi women attending fitness clubs in Riyadh, Saudi Arabia.

Variable	All	Body Image		*p*-Value^2^
		Satisfied	Dissatisfied^1^	
**Sleep duration (%)**				0.499
Insufficient sleeper (<7 h)	224 (54.4%)	26 (11.6%)	198 (88.4%)	
Sufficient sleeper (≥7 h)	188 (45.6%)	26 (13.8%)	162 (86.2%)	
Average sleep duration (hours/day)-mean ± SD	6.70 ± 1.57	7.09 ± 1.45	6.65 ± 1.58	0.056
**Screen time (%)**				0.790
Low Screen Time (<2 h/day)	44 (10.7%)	5 (11.4%)	39 (88.6%)	
High Screen Time (≥2 h/day)	368 (89.3%)	47 (12.8%)	321 (87.2%)	
Average screen time (hours/day)-mean ± SD	4.46 ± 2.21	4.13 ± 1.87	4.51 ± 2.26	0.247
**Food Frequency Intake – mean ± SD**				
Breakfast intake (day/week)	4.6 ± 2.7	4.9 ± 2.7	4.6 ± 2.7	0.388
Vegetables intake (day/week)	4.7 ± 2.1	5.0 ± 1.9	4.7 ± 2.2	0.412
Fruit intake (day/week)	3.7 ± 2.3	4.0 ± 2.4	3.7 ± 2.3	0.350
Milk and dairy products intake (day/week)	4.7 ± 2.3	4.9 ± 2.3	4.7 ± 2.3	0.416
Sugar-sweetened drinks intake (day/week)	1.5 ± 1.8	1.2 ± 1.4	1.6 ± 1.9	0.139
Fast foods intake (day/week)	1.6 ± 1.4	1.5 ± 1.2	1.6 ± 1.4	0.675
French fries/potato chips intake (day/week)	1.4 ± 1.4	1.0 ± 1.1	1.4 ± 1.4	0.087
Cake/donuts intake (day/week)	1.9 ± 1.8	1.5 ± 1.5	2.0 ± 1.9	0.123
Chocolates/candy intake (day/week)	2.7 ± 2.2	2.5 ± 2.2	2.7 ± 2.2	0.465
Energy drink intake (day/week)	0.1 ± 0.5	0.2 ± 0.8	0.1 ± 0.5	0.056
**Classification of PA Category (%)**				0.417
High activity time (≥150 min/week)	425 (92.4%)	48 (12.4%)	340 (87.6%)	
Low activity time (<150 min/week)	35 (7.6%)	4 (15.4%)	22 (84.6%)	
**Sum of activity energy expenditure in METs-min/week–mean ± SD**				
Moderate intensity PA	1560.2 ± 1387.9	1684.1 ± 1618.0	1542.4 ± 1353.3	0.492
Vigorous intensity PA	2242.0 ± 2162.9	2840.1 ± 2623.7	2156.1 ± 2078.6	0.033
Sum from all PA	3802.2 ± 2743.7	4524.1 ± 3358.5	3698.5 ± 2633.0	0.042

*^1^Desired shape < perceived shape. ^2^Chi Squares tests for the differences in proportions, *T*-test for comparison of means (SD).*

Table 3 presents the partial correlation coefficients between perceived BI, desired BI, and BD with selected lifestyle behaviors, while controlling for age. Few correlations are significant. Perceived BI significantly correlated with screen time (*r* = 0.108, *p* = 0.032), chocolates/candy intake (*r* = −0.104, *p* = 0.040), and energy drinks intake (*r* = −0.102; *p* = −0.102, *p* = 0.043). Desired BI significantly correlated with vigorous PA (*r* = −0.138, *p* = 0.020). Finally, BD correlated with chocolate/candy intake (*r* = −0.124, *p* = 0.015) and energy drinks intake (*r* = −0.143, *p* = 0.004).

**TABLE 3:** Partial correlation coefficients (controlling for age) between perceived body image (BI), desired body image (BI), and body dissatisfaction with selected lifestyle behaviors.

Variable	Perceived BI		Desired BI		Body Dissatisfaction	
	*r*	*p*-Value	*r*	*p*-Value	*r*	*p*-Value
Sleep duration (hour/day)	−0.011	0.832	−0.053	0.297	0.000	0.998
Screen time (hour/day)	0.108	0.032	0.031	0.547	0.077	0.130
**Physical activity (METs-min/week)**						
Moderate Physical Activity	0.051	0.311	0.028	0.580	0.042	0.404
Vigorous Physical Activity	−0.118	0.020	−0.138	0.006	−0.043	0.402
Total Physical Activity	−0.066	0.190	−0.094	0.062	−0.012	0.812
**Food frequency intake (day/week)**						
Breakfast intake	−0.070	0.166	−0.035	0.491	−0.060	0.237
Vegetables intake	−0.046	0.369	−0.039	0.439	−0.028	0.578
Fruit intake	−0.025	0.623	−0.006	0.911	−0.011	0.835
Milk and dairy products intake	0.010	0.840	−0.031	0.544	0.036	0.480
Sugar-sweetened drinks intake	0.003	0.960	0.050	0.326	−0.025	0.624
Fast foods intake	−0.044	0.385	−0.019	0.708	−0.052	0.301
French fries/potato chips intake	0.002	0.964	0.002	0.962	0.006	0.911
Cake/donuts intake	−0.076	0.132	−0.059	0.246	−0.050	0.328
Chocolates/candy intake	−0.104	0.040	−0.010	0.847	−0.124	0.015
Energy drinks intake	–0.102	0.043	0.027	0.589	−0.143	0.004

Multivariable logistic regression analysis revealed four variables were predictors of BD (Table 4). Increases in BMI were predictive of increased likelihood of BD (OR: 1.73, CI: 1.38–2.16) while a lower odds of BD was found with consumption of dairy products (OR: 0.79, CI: 0.643–0.980) and energy drinks (OR: 0.26, CI: 0.099–0.679). In addition, club membership of 1–2 years reduced the likelihood of BD (OR: 0.27, CI: 0.078–0.948) as compared to less than 1 year of membership. Other dietary variables, PA, screen time, average sleep duration, and accuracy of BIP were not predictive of BD.

**TABLE 4:** Results of multivariable logistic regression for predictors of body image dissatisfaction from lifestyle variables, body size perception, and demographic factors while controlling for age, education level, family income, and father’s and mother’s education levels.

Dissatisfied^∗^	aOR	CI	*p*-Value
Age	0.98	0.29–3.28	0.975
Education level	0.765	0.21–2.73	0.679
**Family income**			
>25,000 SR^∗∗^	1.00		
15,001–25,000 SR	1.19	0.309–4.576	0.801
15,000 or less	0.70	0.208–2.367	0.569
**Father’s education**	0.86	0.29–2.551	0.786
**Mother’s education**	1.47	0.50–4.330	0.487
**BMI**	1.73	1.38–2.16	<0.001
**Club membership**			
Less than 1 year^∗∗^	1.00		
1–2 years	0.27	0.078–0.948	0.041
**Consumption of dairy products/week**	0.79	0.643–0.980	0.032
**Consumption of energy drinks/week**	0.26	0.099–0.679	0.006

*^∗^Reference category is: satisfied. ^∗∗^Reference group. aOR, adjusted odds ratio; CI, confidence intervals.*

## Discussion

The present study evaluated BI through both a perceptual dimension, BIP, and an attitudinal dimension, BD, in a sample of Saudi women attending fitness centers in the capital of Saudi Arabia. This sample had a high proportion of overweight/obese women with high discrepancy between perceived and desired BI. The majority of the participants dissatisfied with their body and desiring a smaller shape. Those satisfied and dissatisfied with their body shape did not differ significantly in their socio-demographic variables, sleep duration or screen time. Vigorous PA was significantly greater among those who were satisfied. However, when adjusting for socio-demographic variables, multivariable logistic regression analysis revealed significant associations of body shape dissatisfaction with higher BMI, shorter membership duration of fitness club, and reduced dairy products and energy drinks consumption.

Our findings showed that the majority (63%) of our participants are considered in the overweight or obese category. Such rate is similar to the prevalence of overweight plus obesity (61.5%) for Saudi women 15 years and older reported in a recent national survey (Memish et al., 2014). It is therefore not surprising that a relatively high proportion (47%) of the participants misperceived their body shape. This is however similar to most studies that have found underestimation of body weight to be common among adults, particularly those who are overweight/obese (Dorsey et al., 2009; Herman et al., 2013; Clark et al., 2017). In Saudi Arabia, a study of over 900 female university students found 63% of women with overweight/obesity underestimated their actual BMI by 8.5% (−2.5 ± 2.5 kg/m^2^) (Albeeybe et al., 2018). As 92% of our participants reported over 150 min/week of PA, another possible explanation is that studies have found overweight/obese individuals may be more likely to underestimate their weight if they are physically active. Among over 4,000 adults screened for stroke risk, obese subjects who exercised sufficiently showed greater odds of inaccurately perceiving themselves as normal weight as compared to those who were sedentary (OR = 0.45, CI = 0.35–0.57) (Miller et al., 2008). Likewise, a large study of adult Danes found overweight subjects with higher levels of leisure-time PA were twice as likely to misperceive their weight in comparison with sedentary individuals (Matthiessen et al., 2014). Inaccurate perceptions of body size are common, particularly among children and adolescents (Hsu et al., 2016; Neves et al., 2017; López and Rodríguez Cabeo, 2020) and adults (Monteagudo et al., 2015; Sonneville et al., 2016). It was also shown that both over and underestimation to negatively affect weight management and PA in adolescents (Jankauskiene and Baceviciene, 2019) and adults (Voelker et al., 2015). However, in a review of large number of studies on weight perception and associated weight management, it was concluded that perceived overweight was not reliably associated with increased PA or healthy eating and was rather associated with greater disordered eating in some groups (Haynes et al., 2018). On the other hand, accurate estimation of excess weight was observed to have a positive effect on weight management in a 13-year follow-up among female obese adolescents (Lynch et al., 2009).

This study found a high proportion of women with dissatisfaction of body shape (87%) even among normal weight respondents. Previous studies showed that BD is common, particularly among females, both in adolescence and adulthood (Yager and O’Dea, 2008; Fan and Jin, 2015; Voelker et al., 2015; Allen and Walter, 2016; Neves et al., 2017). Similar results were found among Spanish adults (*n* = 1,081), with 67.1% of males and 81.0% females reporting BD (Bibiloni et al., 2017). Likewise, among over 15,000 respondents in the European Union, 54% of males and 69% of females were found to have BD (McElhone et al., 1999).

Among 336 mainly unmarried young adult female university students in the capital, Riyadh, 17% of normal BMI subjects, half of overweight (51%) and the majority of obese subjects (70%) were dissatisfied with their body shape (Alhussaini et al., 2018). Other studies in the Arab world, however, have shown lower rates of BD. Only a third of college females in five Arab countries (Bahrain, Egypt, Jordan, Oman, and Syria) were dissatisfied with their body shape (Musaiger, 2014), however the body weight of subjects was not reported. Likewise, in a study conducted on 368 university students from the East of Saudi Arabia, only a third of females (33.5%) and 21.4% of males were found to have BD, however this may be due to the majority of respondents (65%) having normal BMI (Al-Otaibi et al., 2013).

This study found BMI, period of membership in the gym, and frequency of consumption of dairy products and energy drinks significant predictors for BD. Increased BMI is a consistent predictor of BD among adolescents (Gouveia et al., 2014; Fernández-Bustos et al., 2019; Jankauskiene and Baceviciene, 2019), adults and even children (Neves et al., 2017). Findings from a 10-year longitudinal study in the United States reported a trend among youth of increasing BD as their BMI increased from middle school to young adulthood (Bucchianeri et al., 2013; Feng and Wilson, 2019). Likewise, a longitudinal study of Australian adults found BMI tended to be significantly higher among subjects with BD (Feng and Wilson, 2019). Similarly, data from Netherlands Study of Depression and Anxiety found higher BMI was significantly associated with greater BD independent of depression (Paans et al., 2018).

Body dissatisfaction may be seen as a natural consequence of increased BMI and a precursor for positive health behaviors. It is often proposed that accurate perception of one’s body is vital for motivating weight loss intention and weight loss attempts, particularly among overweight and obese individuals (Baranowski et al., 2003; Rancourt et al., 2017). However, there is strong evidence from longitudinal studies that perceived overweight and BI dissatisfaction are associated with greater weight gain and increased likelihood of overweight/obesity onset over time (Robinson et al., 2015; Haynes et al., 2018), regardless of weight status at baseline and whether the subject’s perception is accurate or inaccurate. Longitudinal studies suggest that BD among overweight adolescents is predictive of negative health consequences including physical inactivity, lower fruit/vegetables intake, eating disorders, and dysfunctional exercise (Neumark-Sztainer et al., 2006; Voelker et al., 2015). On the other hand, healthy weight control behaviors were more prevalent among adolescents with lower BD (Lampard et al., 2016).

In our study, PA was not found to predict BD when adjusted for socio-demographic confounders. However this may be due to the homogeneity of the sample in term of PA levels, as the majority of the women were achieving the recommended levels (≥150 min/week) of weekly PA (Bull et al., 2020). This is contrary to studies showing PA having a positive and direct effect on BD (Fernández-Bustos et al., 2019) as well as other studies that have consistently shown active subjects to have a more positive BI than sedentary subjects (Bibiloni et al., 2017). This may explain why in our sample, females with longer club memberships showed significantly higher BI satisfaction. It is concerning that a high proportion of normal weight women in this sample (68%) were also dissatisfied with their body. Dissatisfaction may trigger disordered exercise behaviors and unnecessary attempts at weight control possibly leading to eating disorders. Secondary analysis of this group, however, did not reveal any significant differences in lifestyle habits with their “satisfied” peers.

The current study showed significant associations of BD with reduced dairy products and energy drinks consumption. Findings from a study conducted on Portuguese girls indicated that after adjusting for all demographic and lifestyle factors, only milk intake was negatively associated with BMI and body fat percentage (Abreu et al., 2012). Proper consumption of milk and dairy products can support body mass satisfaction. Other lifestyle behaviors in the present study did not significantly associate with BD. However, a study conducted among Polish adolescents aged 13–19 years demonstrated that being girls, older participants (17–19 years old), overweight or obese adolescents and having higher screen time (over 4 h) were more likely to be dissatisfied with their body weight (Wawrzyniak et al., 2020).

The present study has several strengths and limitations. Among the strengths of this study is the recruitment of a representative sample of Saudi women from all fitness centers in the city of Riyadh. Second, a reliable and valid lifestyle questionnaire was used that includes PA, sedentary behaviors, sleep, and dietary habits. Third, weight and height of subjects were actually measured and not reported by the participants. On the other hand, some limitations for the present study include the cross-sectional nature of this study which does not allow assumption of causal relationships between the BD, BIP and lifestyle or socio-demographic factors. Second, due to the nature of questionnaires in general, the potential for recall bias cannot be excluded. Third, as the sample came from females who are already participating regularly in fitness centers, it is possible that their BI differs from the general population and thus the study findings may to not be generalized to less active Saudi females. Fourth, BMI was used without accounting for body composition and as this does not distinguish between lean mass and fat mass, we may have misclassified some women who were more muscular. Fifth, the selected BI measures in the present study were based on FRS, which provides a partial assessment of women’s body size concerns.

## Conclusion

The present study examined the associations of BI perception and BD with several lifestyle and socio-demographic factors among Saudi women attending fitness centers in Riyadh. The majority of women were overweight or obese, however, nearly half of the sample inaccurately perceived their body shape, mainly underestimating it. The majority of respondents were also dissatisfied with their body shape, even among those in the normal weight category. In addition, among all investigated lifestyle behaviors, only vigorous and total activity energy expenditure in METs-min/week showed significantly lower values for dissatisfied women compared to satisfied participants. However, when adjusting for socio-demographic variables, logistic regression analysis revealed significant associations of BD with higher BMI, shorter membership duration of fitness club, and reduced dairy products and energy drinks consumption. Other lifestyle behaviors did not significantly associate with BD. The results support previous studies which suggest that accuracy of body shape estimation and BD may not be positively associated with healthy lifestyle habits and weight control behaviors. Our results on the associations between BD and lifestyle behaviors add to those findings from limited studies in the literature. The present findings can inform healthcare providers when intervention strategy is implicated for females with BD, by recognizing the complexity of the associations between BD and lifestyle behaviors. Future studies should compare associations of BD with lifestyle behaviors between males and females attending fitness centers and seeking weight loss.

## Data Availability Statement

The raw data supporting the conclusions of this article will be made available by the authors, upon reasonable request.

## Ethics Statement

The studies involving human participants were reviewed and approved by Princess Nourah Bint Abdulrahman University Ethical Committee–IRB Log Number: 18-0222. The patients/participants provided their written informed consent to participate in this study.

## Author Contributions

HA-H, NA, MA, and AA: study concept. NA, HA-H, and LA: statistical analyses. MA, NA, AA, and LA: data collection supervision. NA and HA-H: interpretation of the findings and drafting the manuscript. All authors critically read, revised, and approved the final version of the manuscript.

## Conflict of Interest

The authors declare that the research was conducted in the absence of any commercial or financial relationships that could be construed as a potential conflict of interest.
